# Impact of Chest Pain Protocol Targeting Intermediate Cardiac Risk Patients in an Observation Unit of an Academic Tertiary Care Center

**DOI:** 10.14740/jocmr2441w

**Published:** 2015-12-28

**Authors:** Tariq Yousuf, Hesam Keshmiri, Jeffrey Ziffra, Ankur Dave, Shoeb Hussain, Joy Iskander, Khansa Ahmed, Bela Nand

**Affiliations:** a Advocate Christ Medical Center, Oak Lawn, IL, USA

**Keywords:** Acute care, Acute coronary syndrome, Chest pain

## Abstract

**Background:**

Chest pain (CP) is a frequent cause of emergency room visits in United States and adds a huge financial burden to our healthcare cost. With the addition of observation units, standard CP protocols have shown to decrease length of stay (LOS) and cost per discharge (CPD). We report our experience with the development and implementation of “CP protocol for intermediate cardiac risk patients” and its impact on healthcare resource utilization at our medical center.

**Methods and Results:**

We retrospectively analyzed 30 patients who presented to Advocate Christ Medical Center (ACMC) with CP and were considered to be at intermediate risk for acute coronary syndrome after obtaining IRB approval. Patients were treated with our standardized CP protocol and labeled as “protocol patients”. Our control group consisted of patients with similar demographics and diagnosis but not treated with our CP protocol admitted in the same time period and under our own faculty. This helped remove the bias of different treating attending. Our protocol algorithm consisted of medications, an electrocardiogram (EKG), cardiac troponin I level, and a stress test if indicated. Primary clinical endpoints for this study were LOS in hours and CPD for patients in our protocol group compared to control group. LOS in the protocol group was lower compared to the control but the difference was not statistically significant (P = 0.74). The average CPD in the control group (mean = $13,446) was almost $830 more than the protocol group (mean = $14,276, P = 0.827).

**Conclusion:**

Implementation of standardized protocols for patients with CP has proven to be a cost effective strategy at several institutions across the country. Our study showed a reduction in CPD although not statistically significant. LOS was also reduced but did not meet statistical significance mainly due to our small sample size. Previous studies had demonstrated much larger savings between a protocol-driven group and a non-protocol-driven group. On further analysis of our data, our protocol group contained five patients who underwent invasive diagnostic tests including computed tomography for pulmonary embolism scans which were not present in the control group. This accounted for the small reduction in costs for the protocol group.

## Introduction

Chest pain (CP) is one of the leading causes of hospital admissions and requires a very comprehensive evaluation; however, accomplishing this in a timely and cost effective manner has been a challenge. This has led to the introduction of “observation units” or “short stay units” and “accelerated diagnostic protocols” at many hospitals across the United States. The utility of these accelerated diagnostic protocols has been validated for the “low cardiac risk” patients, but the data on “intermediate cardiac risk patients” are very sparse. We report our experience with the development and impact on healthcare resource utilization at our medical center using our “CP protocol for intermediate cardiac risk patient”.

Patients presenting with the diagnosis of CP continue to be one of the main diagnosis for emergency room visits adding to the healthcare cost in United States. In 2012, there were 6 million annual visits to the emergency room with the chief complaint of CP [1]. The annual financial burden of CP includes an average length of stay (LOS) of 1.8 days and an average overall cost of $13,187 [2].

Using observation units in hospitals to provide care to certain patients can be more efficient than admitting them to the hospital and can result in shorter LOS and lower costs [3]. Studies have demonstrated that a protocol-based approach with serial monitoring of biomarkers and cardiac stress testing to evaluate intermediate risk patients is both safe and cost effective [4]. Although many of these studies were conducted in the emergency department (ED) setting, this study sought to evaluate the cost per discharge (CPD) and LOS of an evidence-based CP protocol for intermediate risk patients in an observation unit. This study tests our hypothesis that using a protocol-based approach to evaluate CP patients who are at intermediate cardiac risk will have a positive effect on our healthcare cost and assist us in delivering high value care.

## Patients and Methods

The aim of this work was to determine the effectiveness of an observation unit in helping reduce the CPD and LOS in patients who presented to the hospital with a chief complaint of CP. We retrospectively investigated patients who presented to Advocate Christ Medical Center (ACMC) for CP that were deemed to be intermediate risk. Patients were assigned, in an equal ratio, to either our standardized protocol group or the control group. The randomization was blocked to ensure ongoing equality of group size. Our study was designed to include 30 patients. All patients were enrolled from ACMC between the dates of February 27, 2013 and January 15, 2015.

**Figure 1:**
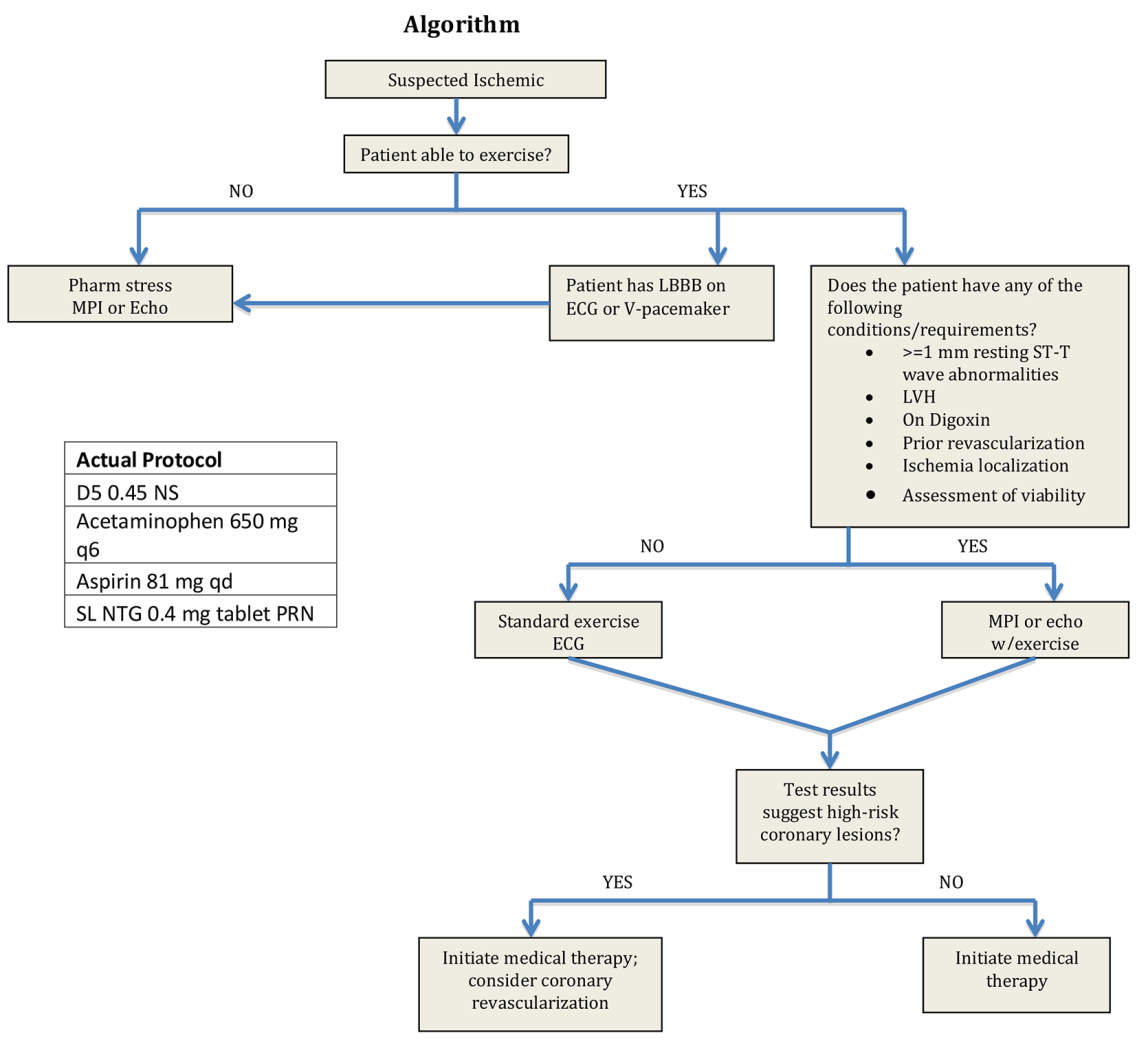
Protocol algorithm.

Our treatment algorithm was initiated upon admission and is listed in Figure 1. Primary clinical endpoints were LOS calculated in hours and CPD for patients in the protocol group versus patients in the control group. Data were collected for this study by means of retrospective chart review. Patient charts were reviewed for inclusion (Table 1) and exclusion criteria (Table 2). Institutional review board approval was obtained prior to data collection.

**Table 1:** Inclusion Criteria

Inclusion criteria
Chest pain
Normal baseline EKG
Negative cardiac enzymes
Atypical angina Sx
< 3 cardiac risk factors

**Table 2:** Exclusion Criteria

Exclusion criteria
TIMI risk ≥ 5
Continued or unrelieved CP
New LBBB on EKG
New transient ST deviation on EKG
New TWI in precordial leads of EKG
Hemodynamic instability
Altered mental status
Elevated cardiac enzymes
Individuals who are not yet adults

Categorical variables are summarized with frequencies and percentages and continuous variables are summarized with means and standard deviations (SD). Analysis groups were non-protocol and protocol. Between-group analysis was performed with independent samples *t*-tests for the primary outcomes of cost and LOS as well as for age. Between-group differences for patient demographic variables, with the exception of age, were performed with Chi-square analysis. Analysis was performed using SPSS 22^®^ (Chicago, IL) and statistical significance was determined at P < 0.05.

## Results

Patient characteristics are presented in Table 3. The majority of the sample patients were female (56.6%) with the mean age of 56.6 ± 10.3. Approximately half of the sample patients were smokers (46.7%) and had hyperlipidemia (46.7%). More than half of the sample patient population had hypertension (63.3%) and about one-fourth had diabetes (26.7%). There was no group difference for gender, current smoker, hyperlipidemia and hypertension (P-values range from 0.13 to 1.00). The control group of patients did have more subjects with diabetes (n = 7, 46.7%) than the protocol group (n = 1, 6.7%, P = 0.04).

**Table 3:** Patient Demographics for Total Sample

	Total sample (n = 30)	Non-protocol (n = 15)	Protocol (n = 15)	P
Gender (female)	17 (56.7)	10 (66.7)	7 (46.7%)	0.27
Current smoker (yes)	14 (46.7)	8 (53.3)	6 (40.0)	0.46
Hypertension (yes)	19 (63.3)	12 (80)	7 (46.7%)	0.13
Hyperlipidemia (yes)	14 (46.7)	7 (46.7%)	7 (46.7%)	1.0
Diabetes (yes)	8 (26.7)	7 (46.7%)	1 (6.7%)	0.04
Stress test performed (yes)	26 (86.7)	11(73.3)	15 (100)	0.10
Negative stress test (if stress test performed)*	25 (96.2)			
Age, mean (SD)	56.6 (10.3)	60.3 (9.80)	52.9 (9.70)	0.05

Values represent N (%) unless otherwise indicated. *Between-group analysis was not performed for this variable because having a stress test was part of the intervention in the protocol group.

Four subjects in the protocol group did not have a stress test and 25 of the 26 who did have a stress test had negative results (96.2%). Between-group analysis was not performed for this variable because having a stress test was part of the intervention in the protocol group.

**Figure 2:**
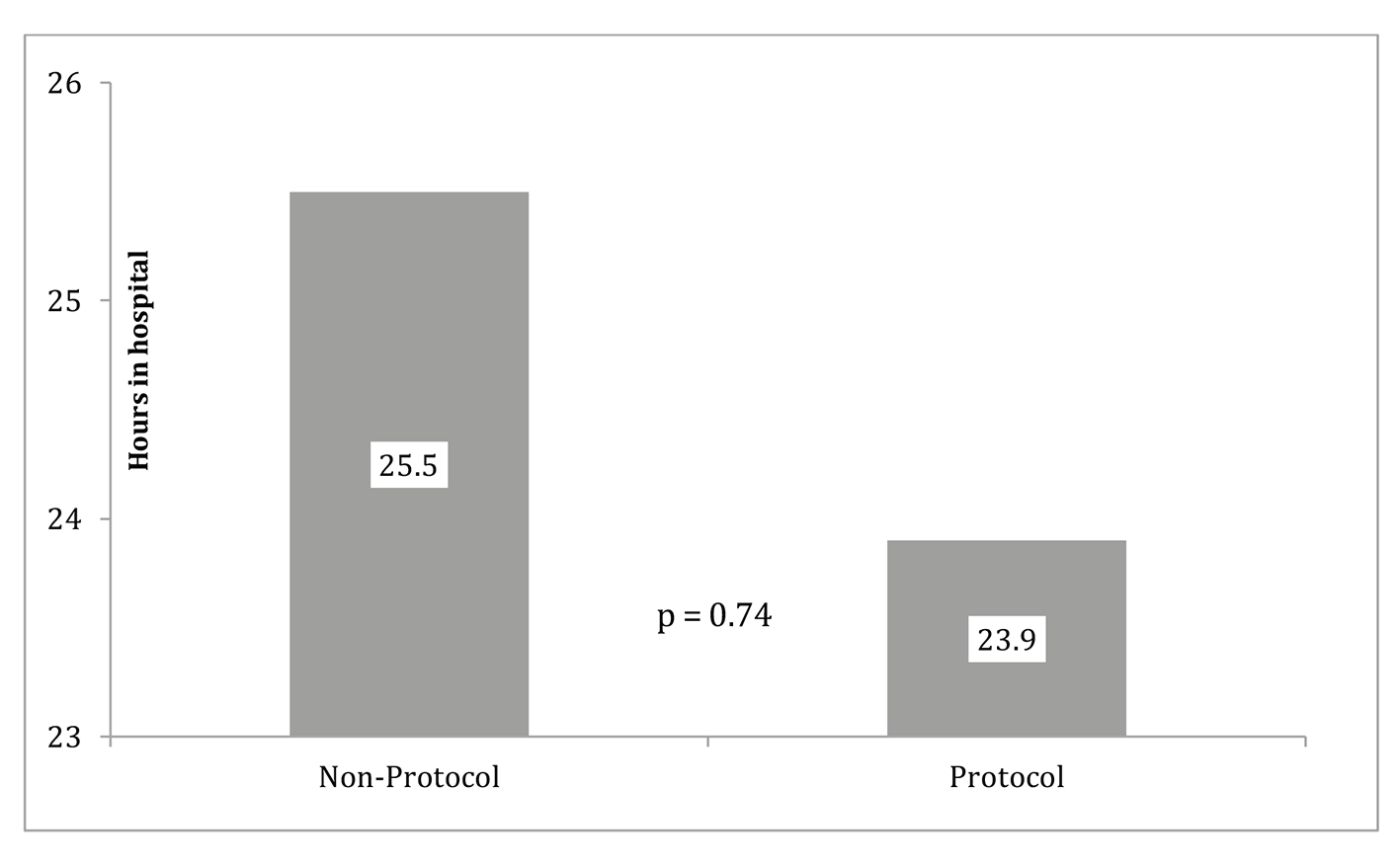
Protocol and non-protocol LOS.

The primary outcomes (LOS and CPD) are displayed in Figures 2 (LOS), 3 (outliers excluded CPD) and 4 (original CPD).

**Figure 3:**
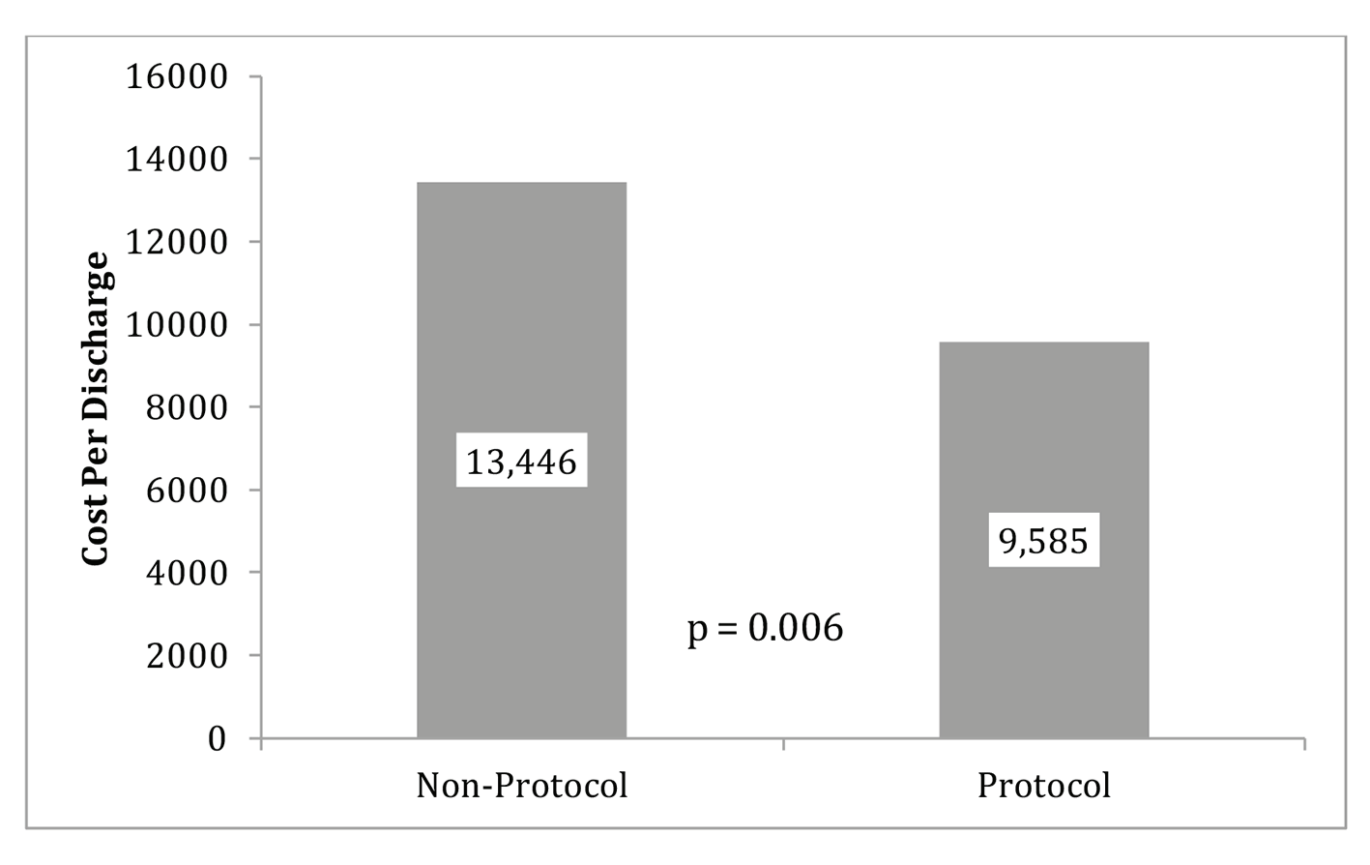
Protocol and non-protocol mean CPD with outliers excluded. Note: five patients were removed from the protocol group due to outliers costs (CTPE) and four subjects in the non-protocol group did not have cost data available. Therefore, the sample sizes for this analysis were 10 for the protocol group and 11 for the non-protocol group.

**Figure 4:**
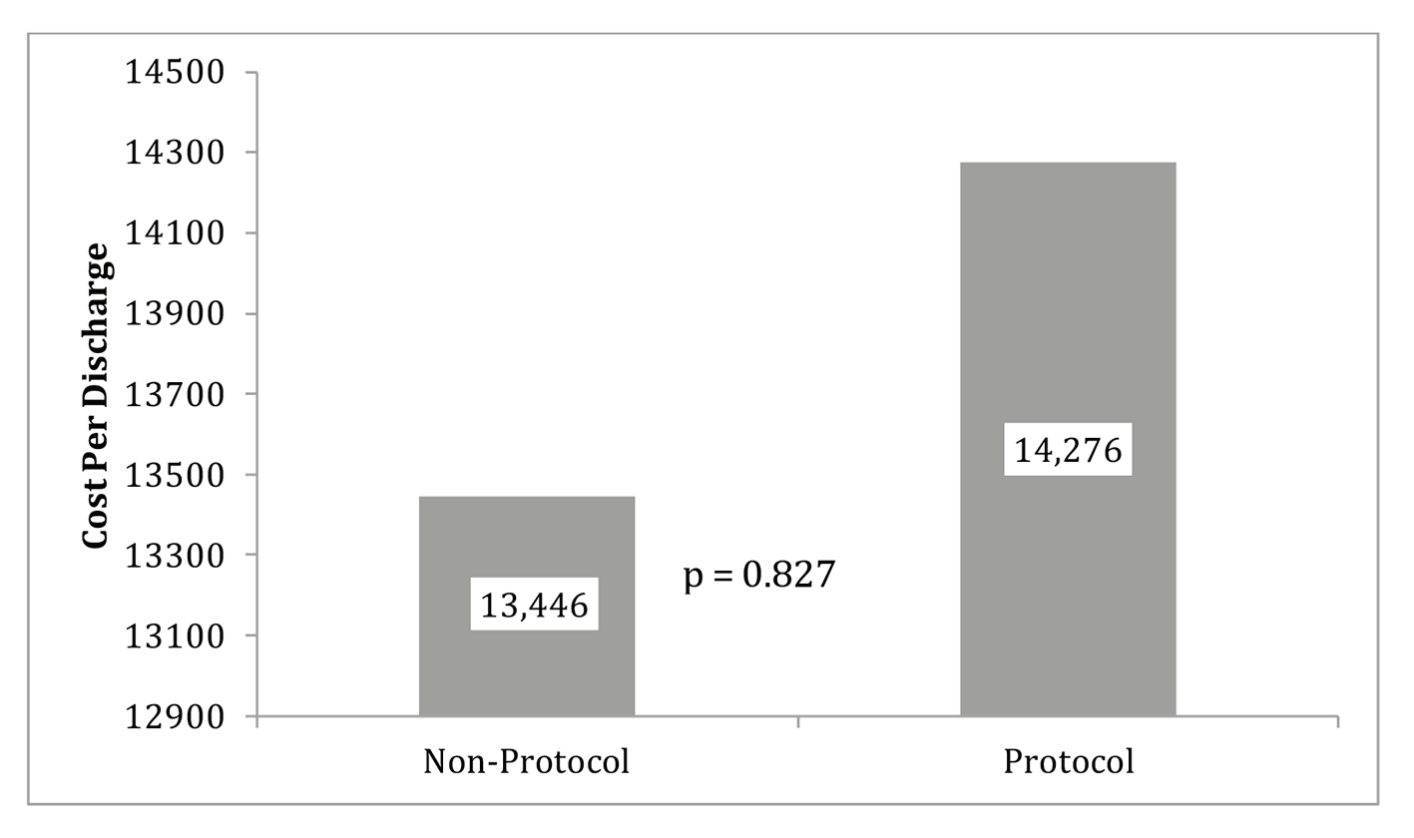
Protocol and non-protocol mean CPD with original data.

Financial data were not available for four subjects in the non-protocol group and five patients in the protocol group had significant interventions imposed by the ED so these patients were excluded as outliers.

The original data showed the average CPD in the control group (mean = $13,446) was almost $830 more than the protocol group (mean = $14, 276). The reduction of CPD in the protocol group was seen with a P-value of 0.837 (Fig. 4). LOS was 23.854 h (SD = 16.6 h) for the protocol group and 25.5 h (SD =10.0 h) for the control group. This reduction of LOS in the protocol group of greater than 1 h was seen with a P-value of 0.741. With outliers excluded, the average cost in the control group (mean = 13,446.1, SD = 2661.3) was almost $4,000.00 more than the protocol group (mean = 9,585.9, SD = 3,013.2) and that difference was statistically significant (P = 0.006) (Fig. 3).

## Discussion

In our study, we evaluated patients that presented with a chief complaint of CP that were deemed intermediate risk for coronary artery disease (CAD). The patients included in our study had a normal baseline electrocardiogram with no ischemic changes, negative cardiac enzymes, atypical anginal symptoms and less than three cardiac risk factors. Low risk patients were classified as patients with a thrombolysis in myocardial infarction (TIMI) risk score of 3 - 4. The prognostic variables are: age greater than 65, more than three traditional CAD risk factors, documented CAD with greater than 50% diameter stenosis, ST-segment deviation, greater than two anginal episodes in the past 24 h, aspirin use in the past 7 days and elevated cardiac biomarkers (CK-MB or Trop). Low risk patients are classified by having a TIMI score of less than 2 and high risk patients have a TIMI score greater than 4.

The concept of CP evaluation in an observation unit has already been performed and proven to reduce CPD and LOS [3]. This study showed reduced in-hospital evaluation period from greater than 2 days to less than 1 day along with cost saving benefit of $1,744.37. The study conducted by Jibrin et al in Maryland looked at low, intermediate and high risk patients and also used a multimodality stress testing protocol which allowed the clinician to order a wide variety of tests based upon patient characteristics.

In a systematic review of the literature performed by Baugh et al, they found an average savings of $1,572 per observation unit visit compared to an inpatient admission. These findings were extrapolated to yield a $3.1 billion savings if observation units were maximized nationally [4]. Goodacre et al analyzed the utility of a CP observation unit in a British hospital and found that there is an opportunity for tremendous savings in these hospitals with the establishment of appropriate cardiac observation units [5]. For hospitals without designated cardiac observation units, implementing a CP protocol in general observation units may elicit the same results as elicited by our study.

We used our intermediate CP protocol to compare primary and secondary outcomes with a control group. In our study, five patients from the protocol group were found to have significant interventions imposed by the ED prior to admission to the observation unit. The significant interventions included a computed tomography for pulmonary embolus (CTPE) to exclude a PE. When these outliers are excluded, CPD and LOS were shown to be reduced in the protocol group. With outliers excluded, the average cost in control group (mean = 13,446.1, SD = 2661.3) was almost $4,000.00 higher than the protocol group (mean = 9585.9, SD = 3013.2) and that difference was statistically significant (P = 0.006). A reduction was seen in LOS in our protocol group but was not statistically significant despite removal of the outliers as stated above. We believe that increasing the power in our study would likely help elucidate greater difference in our clinical endpoints.

### Conclusion

Implementation of standardized protocols for patients with CP has proven to be a cost effective strategy at several institutions across the country for low risk patients. Very few studies have been conducted with a target patient population with a TIMI score of 3 - 4 which qualifies as intermediate cardiac risk. Our study showed a reduction in CPD although not statistically significant. LOS was also reduced but did not meet statistical significance mainly due to our small sample size. Previous studies by Baugh and Goodacre had demonstrated much larger savings between a protocol-driven group and a non-protocol-driven group. On further analysis between the two groups, our protocol group contained five patients who underwent invasive diagnostic tests including CTPE which was not present in the control group. This accounted for the small reduction in costs for the protocol group. Analyzing the costs with outliers removed showed great reductions in costs that did meet statistical significance. This is a promising step toward delivering high value care. More investigation with a larger sample size needs to be conducted using our protocol to confirm these findings.
